# Non-auditory, electrophysiological potentials preceding dolphin biosonar click production

**DOI:** 10.1007/s00359-017-1234-0

**Published:** 2017-12-08

**Authors:** James J. Finneran, Jason Mulsow, Ryan Jones, Dorian S. Houser, Alyssa W. Accomando, Sam H. Ridgway

**Affiliations:** 1 US Navy Marine Mammal Program, Space and Naval Warfare Systems Center Pacific Code 71510, 53560 Hull St., San Diego, CA 92152 USA; 2 National Marine Mammal Foundation, 2240 Shelter Island Dr. #200, San Diego, CA 92106 USA

**Keywords:** Dolphin, Biosonar, Sound production, Auditory brainstem response, Electromyography

## Abstract

The auditory brainstem response to a dolphin’s own emitted biosonar click can be measured by averaging epochs of the instantaneous electroencephalogram (EEG) that are time-locked to the emitted click. In this study, averaged EEGs were measured using surface electrodes placed on the head in six different configurations while dolphins performed an echolocation task. Simultaneously, biosonar click emissions were measured using contact hydrophones on the melon and a hydrophone in the farfield. The averaged EEGs revealed an electrophysiological potential (the pre-auditory wave, PAW) that preceded the production of each biosonar click. The largest PAW amplitudes occurred with the non-inverting electrode just right of the midline—the apparent side of biosonar click generation—and posterior of the blowhole. Although the source of the PAW is unknown, the temporal and spatial properties rule out an auditory source. The PAW may be a neural or myogenic potential associated with click production; however, it is not known if muscles within the dolphin nasal system can be actuated at the high rates reported for dolphin click production, or if sufficiently coordinated and fast motor endplates of nasal muscles exist to produce a PAW detectable with surface electrodes.

## Introduction

**Fig. 1:**
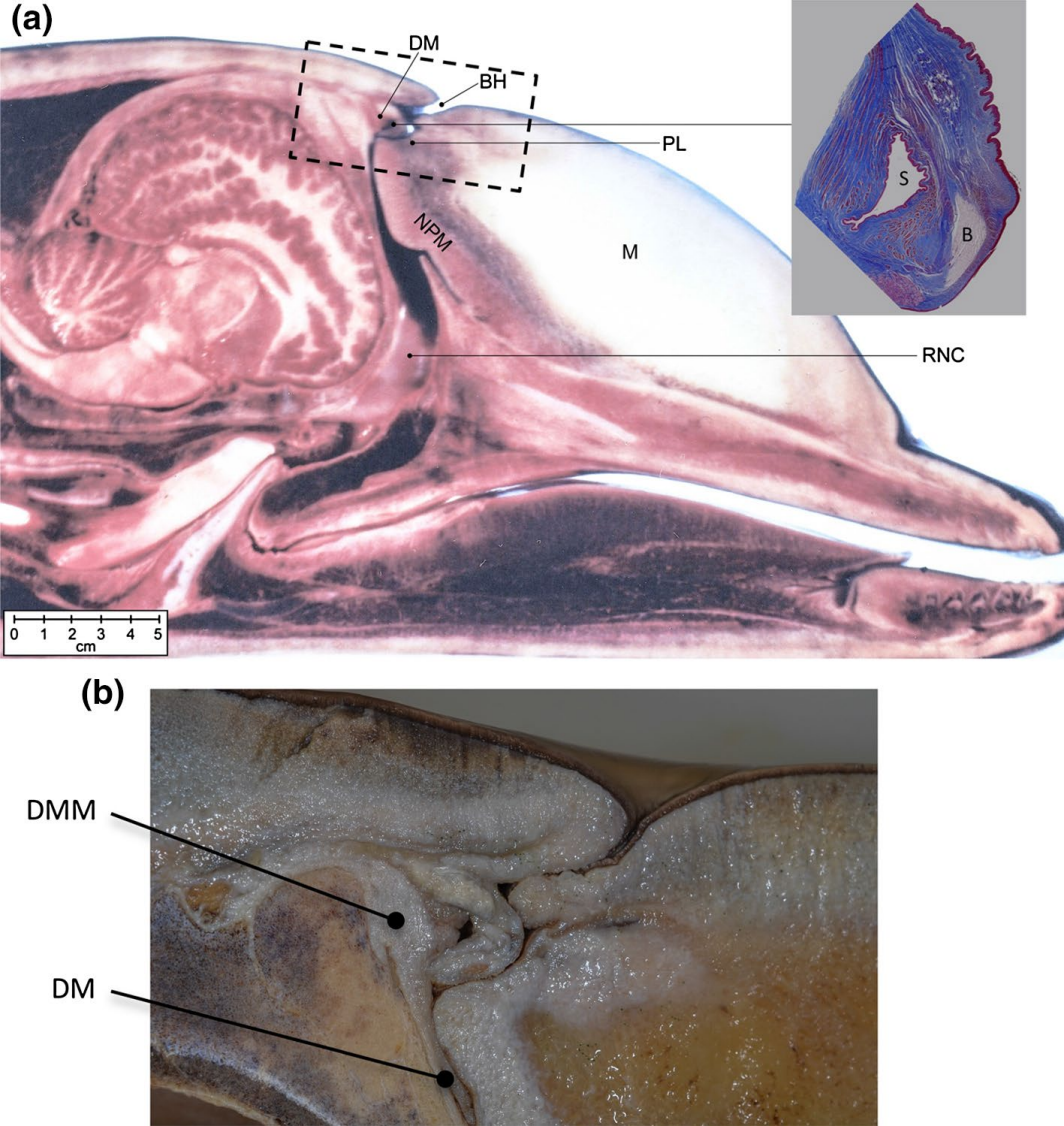
**a** View of sagittal section of the head of an immature dolphin. The detail at right shows an expanded view of the area of the posterior nasofrontal sac (S) and bursa of the posterior phonic lip (B) under Masson’s Trichrome stain. **b** Detail view of the phonic lips and diagonal membrane in another dolphin. The approximate location for image **b** is indicated by the dashed rectangle in **a**. *BH* blowhole, *DM* diagonal membrane, *DMM* diagonal membrane muscle, *M* melon, *NPM* nasal plug muscle, *PL* phonic lips, *RNC* right nasal cavity

Dolphins and other odontocetes (toothed whales) possess biological sonar (biosonar) systems, whereby sound pulses (“clicks”) are emitted and returning echoes are analyzed to detect, localize, and identify underwater objects (e.g., during foraging). Dolphins produce the sound pulses required for echolocation by passing pressurized air past the “phonic lips” located within the nasal passages (Fig. 1, Ridgway et al. 1980, 2015; Amundin and Andersen 1983; Ridgway and Carder 1988; Cranford et al. 1996, 2011; Cranford 2000). Sound pulses produced at the phonic lips undergo complex interactions with the skull, air spaces, and other tissues in the head as they are coupled into the melon and emitted into the water in a forward-directed beam (Aroyan et al. 1992; Au et al. 2010; Finneran et al. 2014). Like most other odontocetes, dolphins possess two (left and right) pairs of phonic lips (Cranford 1992, 1999; Cranford et al. 2011); however, echolocation pulses appear to be typically generated using the right phonic lips, which are physically larger than those on the left (Mead 1975; Aroyan et al. 2000; Ridgway et al. 2009; Madsen et al. 2013). Despite considerable progress in identifying the general site and pneumatic nature of click production, the specific mechanisms underlying click production are still poorly understood. For example, the specific vibrating structures responsible for the high-frequency, damped oscillations seen in farfield click recordings have not been identified (Cranford 2000). The extent to which the shape of the melon and air spaces can be manipulated to control beam characteristics is unknown (Harper et al. 2008; Cozzi et al. 2017). Finally, it is not clear if dolphins can control the timing of individual biosonar pulses; i.e., are individual clicks produced via discrete muscular activity, or are trains of clicks produced when air is pressured to the point where it escapes past the phonic lips?

Biosonar clicks produced by odontocetes are audible to the echolocating animal (Bullock and Ridgway 1972; Ridgway and Au 1999). Taking advantage of this, Supin et al. (2003) measured auditory brainstem responses (ABRs) in a false killer whale (*Pseudorca crassidens*) in response to the animal’s own biosonar click (the “self-heard” click) to investigate biosonar automatic gain control mechanisms. A number of subsequent studies utilized similar methodologies to investigate automatic gain control and biosonar signal processing in the same false killer whale (reviewed by Supin and Nachtigall 2013), dolphins (e.g., Li et al. 2011; Finneran et al. 2013, 2017), and porpoises (e.g., Beedholm et al. 2006; Linnenschmidt et al. 2012). In these studies, surface electrodes embedded in suction cups were placed on the animals’ heads and used to record the instantaneous electroencephalogram (EEG) while they echolocated. ABRs to the self-heard click and returning echoes were obtained by synchronously averaging epochs of EEG data time-locked with biosonar clicks.

In addition to ABRs to the self-heard click and returning echoes, a recent study also reported deflections in the averaged EEG occurring around the time of click emission, but before the first waves in the self-heard click ABR (Finneran et al. 2016). These early deflections in the time-locked, averaged EEG are referred to here as the “pre-auditory wave” (PAW). Finneran et al. (2016) reported that the PAW was smaller when the non-inverting electrode was moved caudally away from the blowhole; however, investigation of the PAW was outside the scope of the study and therefore, no effort was made to identify its underlying cause. The present paper describes experiments that investigate the origin of the PAW and the relationship between the PAW morphology and the anatomical site of click generation.

## Methods

### Experimental task

The experimental approach featured measurements of electrophysiological potentials and click emissions while dolphins performed an echolocation task. Electrophysiological potentials were measured using surface electrodes placed on the head in six different configurations. Click emissions were measured using contact hydrophones placed on the melon (similar to Diercks et al. 1971; Au et al. 2010; Madsen et al. 2010, 2013) and a hydrophone in the biosonar transmission beam acoustic farfield. Tests were conducted in July 2016, within a 9 m × 9 m floating, netted enclosure at the US Navy Marine Mammal Program facility in San Diego Bay, California. Subjects consisted of two bottlenose dolphins (SAY, female, 37 years, ~ 220 kg; TRO, male, 24 years, ~ 180 kg) with upper hearing limits (based on psychophysical threshold measurements) of ~ 140 kHz, which is “normal” for a bottlenose dolphin (Johnson 1967; Erbe et al. 2016).

The echolocation task was a go/no-go, echo-change detection task, where the dolphin was required to produce a conditioned acoustic response (SAY: whistle, TRO: echolocation burst-pulse) after detecting a change in biosonar echoes and to withhold the response otherwise (see below and Finneran et al. 2013, 2016). Echoes were generated using a “phantom” echo generator rather than a physical target (see below and Simmons 1973; Au et al. 1987). Each experimental session lasted approximately 60–90 min and consisted of 120–130 discrete trials. During each trial, the dolphin positioned itself in a hoop (~ 1 m depth, facing outward from the enclosure towards San Diego Bay), produced echolocation clicks, and listened to the returning phantom echoes. On 80% of the trials, the phantom target impulse response changed after a random interval of 5–10 s followed by a 2-s response interval. On the remainder of the trials, the phantom target remained constant for the 7- to 12-s trial duration. Correct responses by the dolphin were rewarded with fish. Sessions were divided into blocks of ten trials within which the contact hydrophone and electrode positions were fixed.

### Signal generation and recording

Two piezoelectric transducers (1089D, International Transducer Corp, Santa Barbara, CA and TC4013, Reson Inc., Slangerup, Denmark) were located approximately 1.3 m in front of the subject’s blowhole when positioned in the hoop. Phantom echoes were produced by capturing the dolphin’s outgoing echolocation clicks with the 1089D hydrophone and convolving the clicks with a target impulse response to create echo waveforms (for details see Finneran et al. 2010, 2013, 2016). Two impulse responses, denoted as A and B, were used to produce echo waveforms. Echo A consisted of a replica of the received click and echo B consisted of a replica of the click whose amplitude “jittered” (i.e., alternated on successive presentations) by 3 dB (± 1.5 dB). The p–p amplitude of echo A relative to the click was − 85 dB, respectively, and the simulated target range was 10 m (i.e., ~ 13-ms echo delay relative to the biosonar click). Analog echo waveforms were filtered (5–200 kHz, 3C module, Krohn-Hite Corporation, Brockton, MA, USA), attenuated (PA5, Tucker-Davis Technologies, Alachua, FL, USA), amplified (7600M, Krohn-Hite Corporation, Brockton, MA, USA), and broadcast to the dolphin using the TC4013 transducer.

**Fig. 2:**
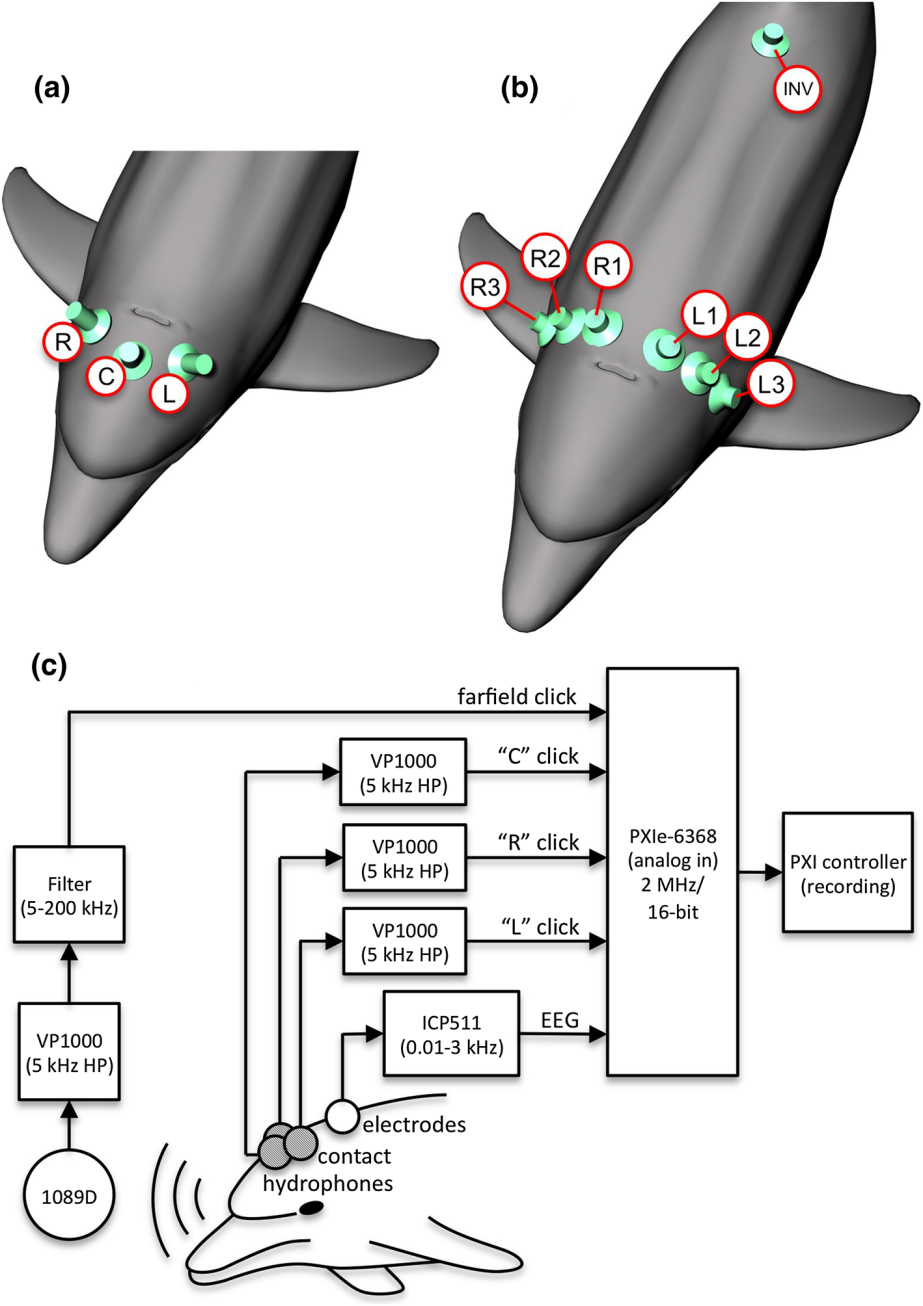
**a** Click emissions were recorded using three contact hydrophones placed on the dolphin’s melon, 7 cm from the blowhole, on the centerline (C) and left (L) and right (R) of the centerline, and a single hydrophone along the main transmit axis in the acoustic farfield. The contact hydrophones at the L and R positions were switched after every ten trials. **b** The instantaneous EEG was based on the potential difference between a non-inverting electrode placed at one of six locations near the blowhole (L1, L2, L3, R1, R2, R3) and an inverting electrode (INV) located near the base of the dorsal fin. **c** Hydrophone and electrode signals were amplified, filtered, digitized and stored to computer disk for later analysis. Time delays associated with the electronic equipment were measured and used to correct the measured latencies for the biosonar clicks and instantaneous EEG

Instantaneous sound pressures associated with biosonar click emissions were measured using four hydrophones: the 1089D hydrophone positioned in the farfield along the primary transmit axis and three contact hydrophones consisting of piezoelectric transducers (TC4013, Reson Inc., Slangerup, Denmark) embedded in suction cups and placed on the melon (Fig. 2a). The contact hydrophone positions were designed as L, C, and R: the C position was on the midline, ~ 7 cm from the blowhole, and the *L/R* positions were at the same distance offset from the midline by approximately ± 60°. Signals from the hydrophones were filtered (see Fig. 2c) using a VP1000 pre-amplifier (5 kHz high-pass, Reson Inc., Slangerup, Denmark). The 1089D was additionally band-pass filtered from 200 Hz to 200 kHz (8-pole Butterworth 3C module, Krohn-Hite Corporation, Brockton, MA, USA). All hydrophone signals were digitized at 2 MHz with 16-bit resolution using a PXIe-6368 data acquisition device (National Instruments, Austin, TX, USA) and streamed to disk for later analysis. To ensure that any differences in contact hydrophone response, construction, or position did not bias the results, the positions of the L and R hydrophones were exchanged after every ten trials.

Electrophysiological potentials were measured using 10-mm gold-cup surface electrodes (Viasys Healthcare) embedded in suction cups and coupled to the skin using conductive paste. Three electrodes were used: the “non-inverting” electrode was located approximately 7 cm caudal to the blowhole at one of six lateral positions (Fig. 2b), the “inverting” electrode was placed on the midline near the base of the dorsal fin, and the ground (common) electrode was placed in the seawater near the dolphin. A biopotential amplifier (ICP511, Grass Technologies, West Warwick, RI, USA) filtered (0.01–3 kHz) and amplified (86 dB) the potential difference between the non-inverting (+) and inverting (−) electrodes. The differential electrode voltage, primarily representing the instantaneous EEG, was digitized at 2 MHz with 16-bit resolution using the PXIe-6368. The position of the non-inverting electrode was moved every 20 trials.

### Analysis

Within each trial, the arrival time of each biosonar click at melon position C was determined and used to estimate the time of click generation, based on the estimated distance between the contact hydrophone and the dolphin’s phonic lips (10 cm) and a nominal sound speed of 1450 m/s. Position C was used, rather than R, because the same hydrophone was always used at position C and the recorded waveform at C showed less variability compared to that at the L and R positions. Individual 30-ms time epochs, beginning 10 ms before click emission, were then identified. For each time epoch, the instantaneous EEG signal was extracted, decimated to a 100-kHz sampling rate, and saved as a separate file (an EEG waveform “clip”). During EEG clip extraction, time intervals containing a whistle or burst-pulse response, epochs corresponding to clicks with inter-click intervals < the two-way travel time between dolphin and phantom target, epochs occurring after target jitter initiation, and epochs with EEG peak amplitude > 40 μV were excluded. Time periods with relatively large dolphin head movements (identified by oscillations in the farfield click amplitude) were also excluded from EEG clip extraction. EEG clips were then grouped by subject and non-inverting electrode position, digitally band-pass filtered (300–3000 Hz, zero-phase 6th-order Butterworth), and synchronously averaged. The high-pass cutoff of 300 Hz was used to remove low-frequency drift in the averaged EEG baseline that made interpreting the ABR/PAW peaks difficult; preliminary analyses showed little effect of the high-pass filter on the ABR or PAW peaks. For visualizing the averaged EEGs, two separate averages were computed, each containing half the total available epochs.

Hydrophone data were analyzed by comparing the times-of-arrival (based on threshold-crossing) at each contact hydrophone for each click recorded on the farfield hydrophone. Time delays associated with the analog electronics (amplifiers and band-pass filters) were estimated by passing signals through the equipment and measuring the resulting time delays. For click measurements, the signals consisted of recorded dolphin clicks. For EEG measurements, 1-kHz tone bursts and digitized waveforms of dolphin click-evoked ABRs were used. The resulting time delays (2 μs for biosonar clicks, 220 μs for the EEG) were then used to correct the latencies of the measured biosonar clicks and EEG epochs.

The performance of each dolphin in the echolocation task was quantified using the hit rate (the number of responses during echo-change trials, divided by the number of echo-change trials) and the false alarm rate (the number of responses during no-change trials, divided by the number of no-change trials).

## Results

The dolphins SAY and TRO participated in 250 and 240 trials, respectively. Hit rates were 89 and 90%, and false alarm rates were 25 and 14% for SAY and TRO, respectively.

**Fig. 3:**
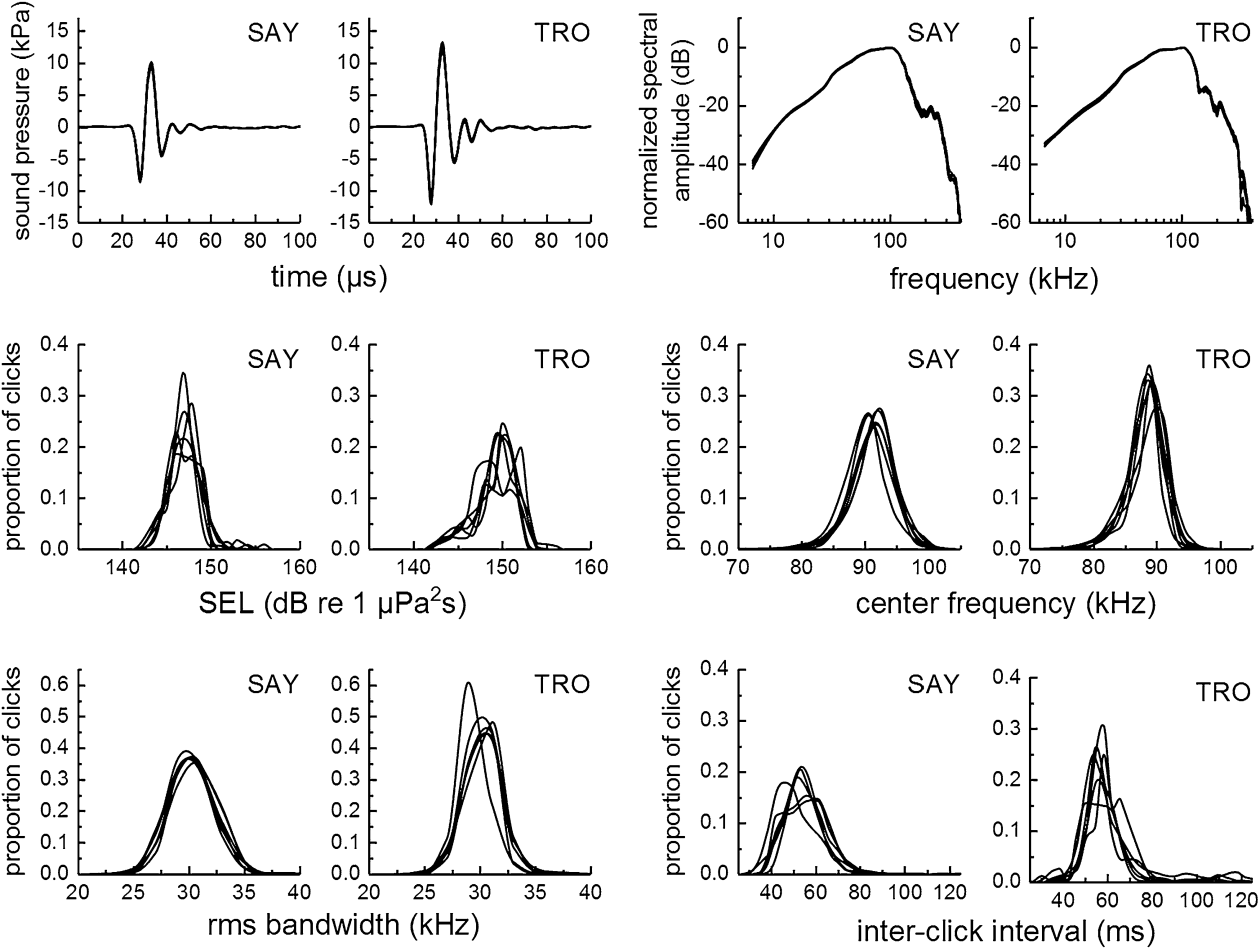
For each subject, properties of biosonar clicks recorded in the farfield along the main transmit axis were similar across trials with the same non-inverting electrode position. In each panel, mean values for the six experimental conditions (L1, L2, L3, R1, R2, R3) are overlaid. Click amplitudes were corrected from the measurement distance of ~ 1.3 m to the source level reference distance of 1 m, assuming spherical spreading loss with no attenuation

A total of 28,510 and 20,789 clicks and EEG epochs were obtained for SAY and TRO, respectively. For each non-inverting electrode location, the number of clicks/epochs ranged from 4184 to 5268 for SAY and 2974 to 4210 for TRO (Table 1). Properties of clicks recorded in the farfield (Fig. 3) were similar across groups of trials with the same non-inverting electrode position (i.e., differences between averaged EEGs with different electrode positions cannot be explained by differences in click emissions). Click instantaneous sound pressures resembled exponentially damped sinusoids with peak pressures of ~ 10 to 15 kPa, p-p source levels (SLs) from ~ 200 to 213 dB re 1 μPa at 1 m, and SELs from ~ 142 to 155 dB re 1 μPa^2^s (all amplitude measures are referenced to 1 m, assuming spherical spreading loss). Center frequencies ranged from 80 to 100 kHz, rms bandwidths were between 25 and 35 kHz, and inter-click intervals varied from 40 to 80 ms for both dolphins. Clicks produced by TRO tended to have higher amplitude, higher frequency content, and larger inter-click intervals compared to those produced by SAY. The correlation between click p–p sound pressure measured with the center contact hydrophone and that measured in the farfield was moderate (SAY: *r* = 0.617; TRO: *r* = 0.553). Correlations between the farfield hydrophone and the *L*/*R* contact hydrophones were relatively low (0.12 < *r* < 0.51). The limited range of click levels (15 dB) and the ability of the animals to move their heads in the hoop likely contributed to the relatively low correlation between clicks measured on the melon and in the farfield.

**Table 1:** Number of clicks and EEG epochs obtained for the dolphins SAY and TRO with the non-inverting electrode at the positions designated L1, L2, L3, and R1, R2, R3 (see Fig. 2)

Subject	Condition	Epochs
SAY	L3	4623
	L2	4619
	L1	4184
	R1	5268
	R2	5208
	R3	4608
TRO	L3	3172
	L2	2974
	L1	3211
	R1	4210
	R2	3412
	R3	3810

**Fig. 4:**
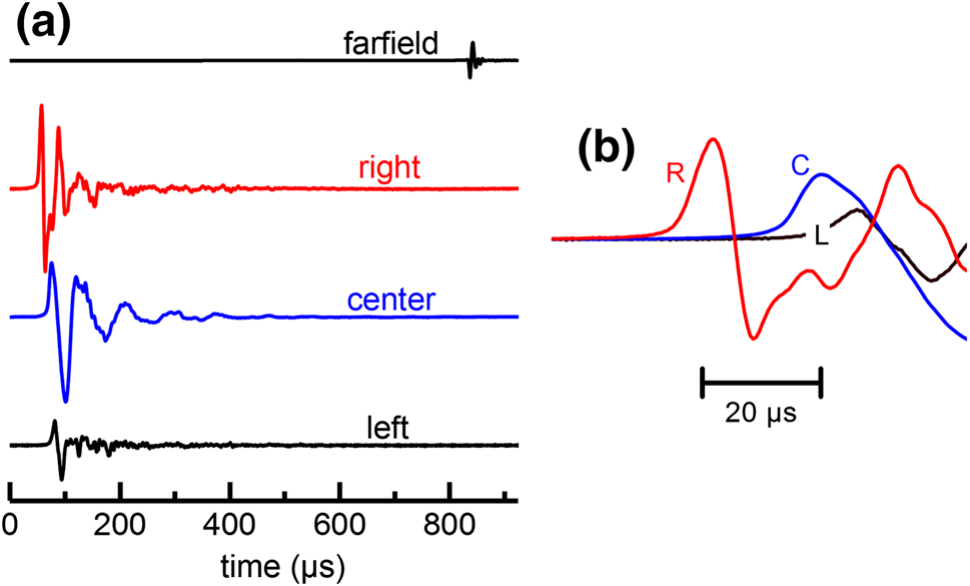
**a** Representative hydrophone data for a single epoch showing timing relationships between the instantaneous sound pressures recorded at the farfield hydrophone and the contact hydrophones located on the left (L), center (C), and right (R) of the melon relative to the midline. The time origin is the estimated time of click generation, based on the estimated distance between contact hydrophone C and the dolphin’s phonic lips and a nominal sound speed of 1450 m/s. **b** For all ~ 49,000 biosonar clicks, the time-of-arrival was shortest for the right contact hydrophone, implicating the right pair of phonic lips as the site of click production

Figure 4 shows a representative example of the instantaneous sound pressure recorded from the contact hydrophones for a single epoch of data. Sound pressures recorded by the *L/R* contact hydrophones (off the main transmit axis) exhibited more complex signatures compared to the center and farfield hydrophones. For all epochs, the biosonar click time-of-arrival was shortest for the right contact hydrophone; i.e., all ~ 49,000 biosonar clicks arrived at the right contact hydrophone first, implicating the right pair of phonic lips as the site of click production for both dolphins.

**Fig. 5:**
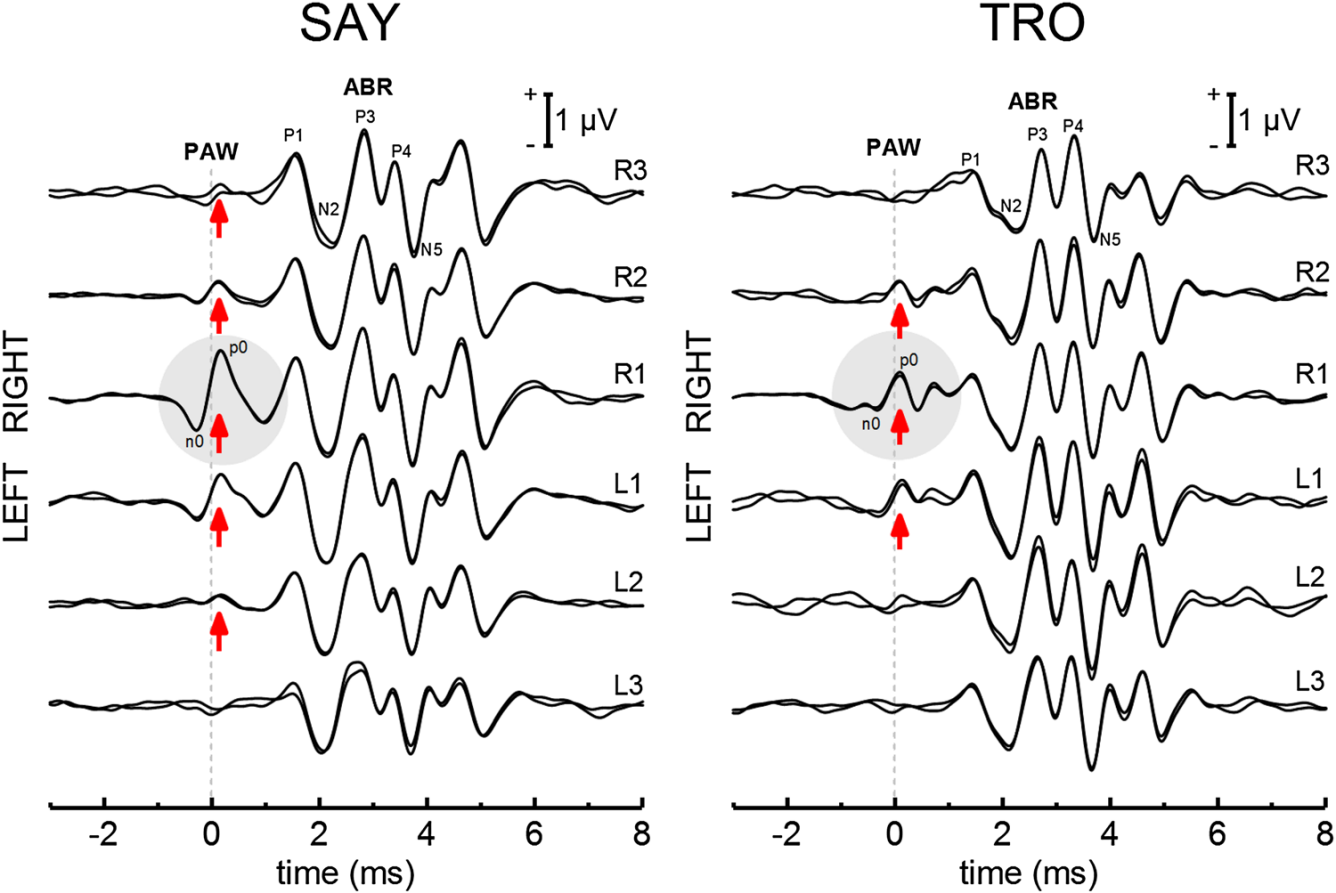
Averaged EEGs for the dolphins SAY and TRO for each of the six non-inverting electrode positions. In each series, the total collection of epochs (see Table 1) is split to create two overlaid waveforms. The time origin is the estimated time of click generation. ABRs to the self-heard click were visible in all records. Early waves in the averaged EEG (the PAW, indicted by the arrows and shaded circle) were visible for some, but not all electrode positions. The PAW consisted of three to five peaks, with the first negative (n0) and positive (p0) peaks being the most prominent. The initial negative deflection of the PAW began ~ 500 μs before click generation. PAW amplitudes were largest at the R1 position, decreased as the non-inverting electrode was moved in either lateral direction, and were asymmetrical towards the right side (i.e., at comparable lateral distances, the PAW was larger on the right)

Figure 5 shows averaged EEGs for SAY and TRO for each of the six electrode configurations, with the total collection of epochs for each condition split to create two overlaid waveforms. ABRs to the self-heard click resembled the typical morphology for dolphins (Ridgway et al. 1981; Popov and Supin 1990) and were visible in all records. Additional waves in the averaged EEG occurred after the N5-peak in the self-heard click ABR and were visible in all records; the waves likely reflect the ABR to the echo from the farfield hydrophone. Early waves in the averaged EEG (the PAW, indicated by the arrows and shaded region in Fig. 5) are visible for some, but not all electrode configurations. The PAW consisted of three to five peaks, with the first negative (n0) and positive (p0) peaks being the most prominent. At larger amplitudes, the duration of each peak was approximately 0.6–0.7 ms and the overall duration was about 2 ms. The initial negative deflection of the PAW began ~ 500 μs before click generation. PAW amplitudes were largest at the R1 position, and decreased as the non-inverting electrode was moved in either lateral direction. The PAW amplitude distributions were asymmetrical and favored the right side (i.e., at comparable lateral distances, the PAW was larger on the right).

**Fig. 6:**
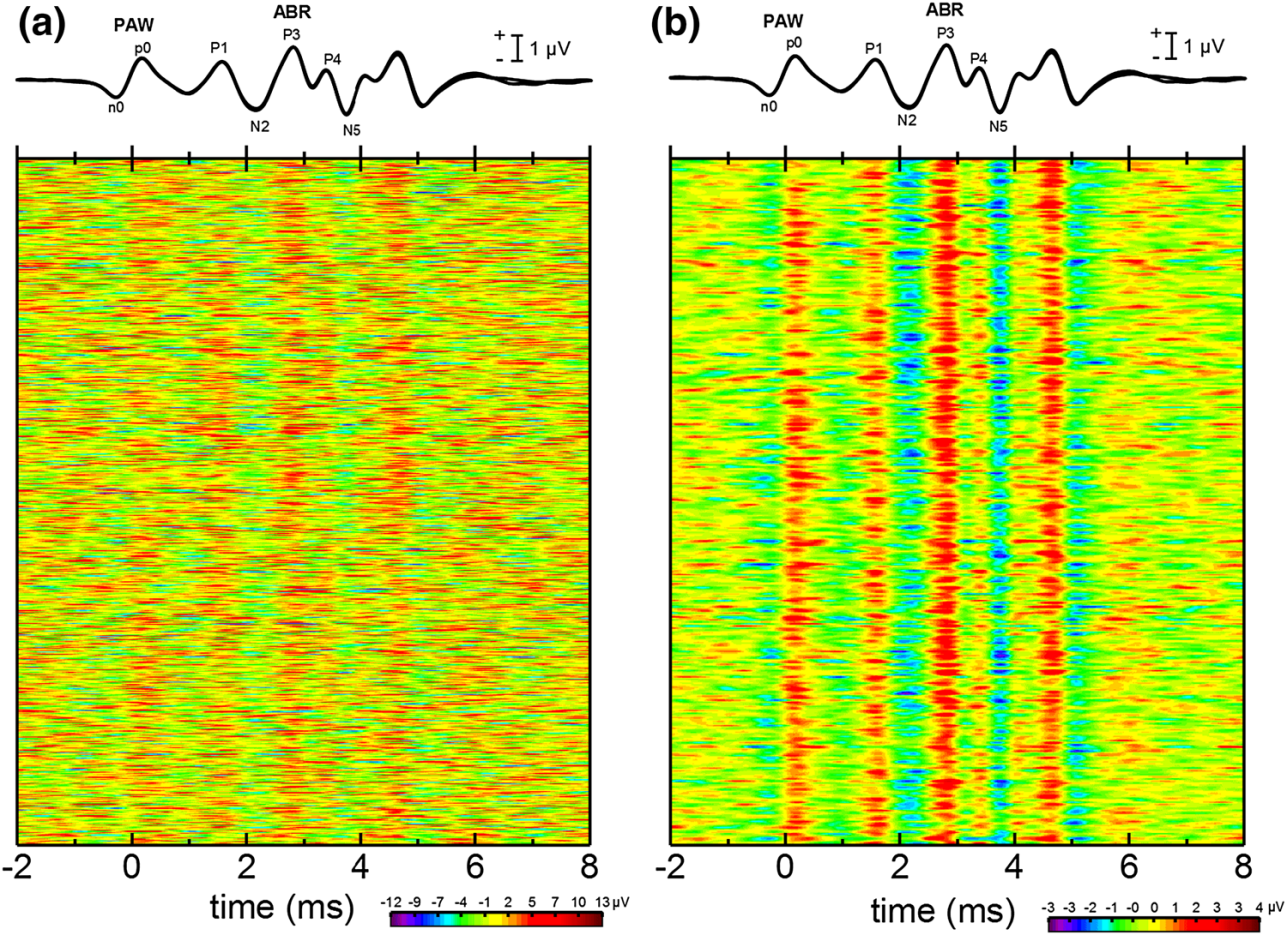
**a** Individual epochs of the instantaneous EEG for the dolphin SAY, with the non-inverting electrode at the R1 position. Each row represents a single epoch, with the amplitude of the instantaneous EEG represented by color. The time origin is the estimated time of click generation. The presence of vertical banding in the image indicates deflections in the instantaneous EEG that are time-locked to click generation. **b** Averaged EEGs for the dolphin SAY, each based on 16 epochs, obtained with the non-inverting electrode at the R1 position. Each row represents the synchronous average of 16 EEG epochs, with the amplitude of the averaged EEG represented by color [i.e., the data from **a** averaged across each 16 rows]. The time origin is the estimated time of click generation. Averaging the EEG reduces the residual background noise and improves the sign-to-noise ratio of time-locked activity in the EEG, which reveals itself as vertical banding. Similar degrees of time-locked activity are visible for the ABR peaks and peaks n0 and p0 of the PAW, suggesting that the PAW is present in each epoch of data, and not the result of large deflections in only a few epochs

It is important for understanding the origins of the PAW to know whether it is present in each epoch of EEG data (the presence of a visible PAW in the averaged EEG does not necessarily mean that the PAW is present in every epoch). Although the SNR of the PAW within a single EEG epoch is too low for the PAW to be identified within the physiological background noise, temporally aligning individual epochs of EEG data, or subaverages composed of only a few epochs of EEG data, can highlight trends across EEG epochs that reveal the presence of time-locked, low SNR signals (see Makeig and Onton 2009). Figure 6a shows the 5268 individual EEG epochs for SAY with the non-inverting electrode at R1, where each row represents an EEG epoch, each column is a time sample, and instantaneous amplitudes are represented by colors. Vertical color patterns—indicating temporally consistent peaks/troughs in the EEG epochs—are visible not only for the ABR peaks P1–N5, but also for the PAW peaks n0 and p0 occurring near the time of click emission. Figure 6b presents the same data, with each row representing a subaverage of 16 EEG epochs. This has the effect of reducing the residual background noise in each subaverage and making the vertical patterns of peaks/troughs more obvious (although this introduces epoch-to-epoch ambiguity). The consistent vertical patterns for peaks and troughs of the PAW in Fig. 6 indicate that the PAW deflections were present in each EEG epoch and not the result of large deflections in only a few epochs.

**Fig. 7:**
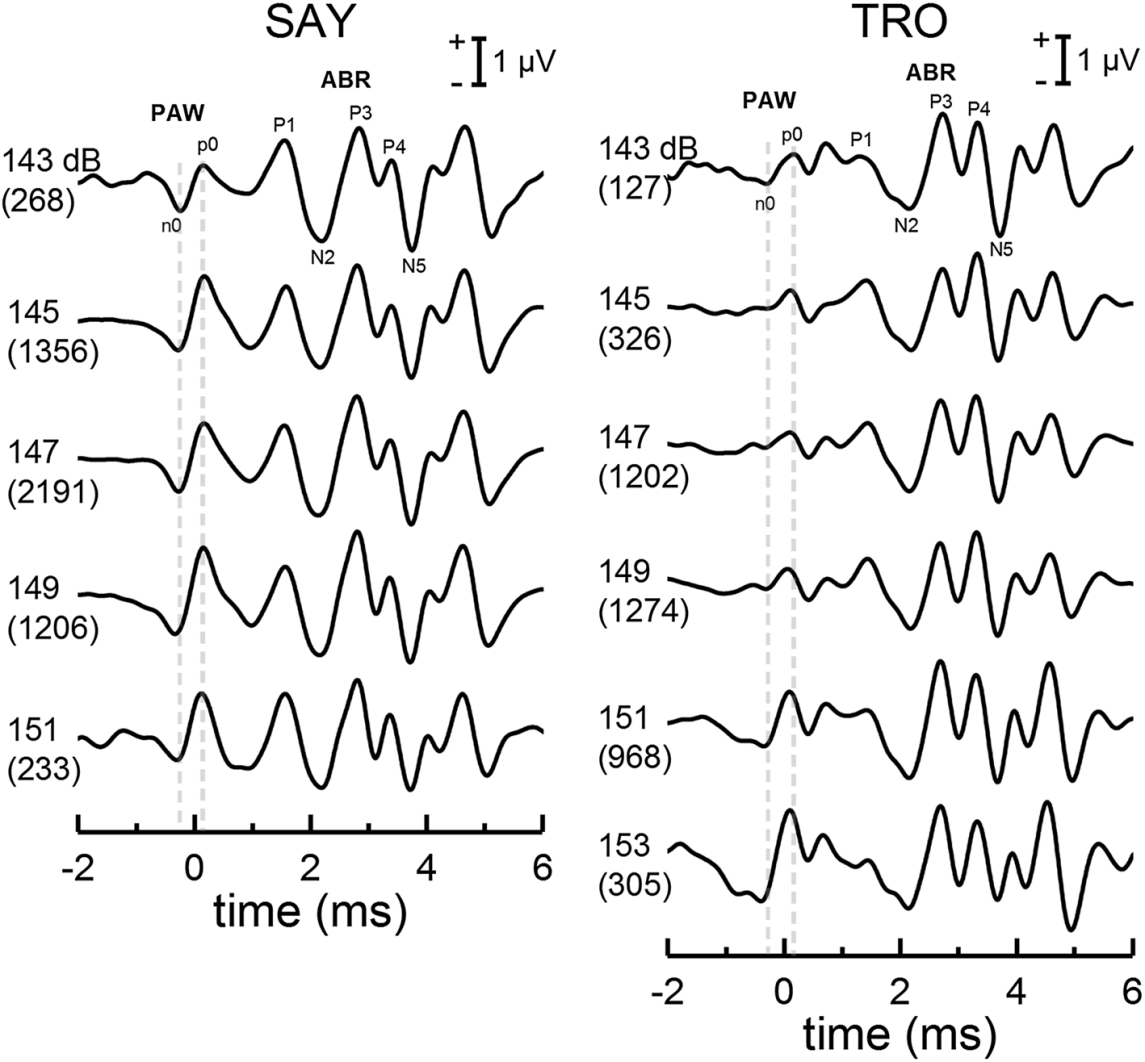
Averaged EEGs for dolphins SAY and TRO created from groups of epochs with farfield click SELs within 2-dB bins. The upper number associated with each series indicates the center SEL of the bin, in units of dB re 1 μPa^2^s. The lower number in parentheses indicates the number of EEG epochs within the average. The PAW is visible in each averaged EEG and the amplitude of peak p0 tended to increase with click SEL; however, given the limited range of SELs and few epochs for some conditions, these data should be interpreted with caution

The data from Fig. 5 with the non-inverting electrode at the R1 position (the condition resulting in the largest PAW) were also analyzed to examine how the PAW amplitude and latency changed with click SEL. When EEG epochs were grouped according to click SEL and averaged, the PAW was also visible (Fig. 7). The amplitude of p0 (the most prominent peak in the PAW) tended to increase with click SEL at a rate of 0.07 μV/dB, while p0 latency tended to decrease with increasing click SEL at a rate of − 4 to − 8 μs/dB. These data should be interpreted with caution due to the limited range of SELs (10–12 dB) for which enough epochs were available to obtain meaningful PAW and ABR estimates (estimated here as ≥ 100 epochs), as well as the potential that some clicks recorded by the farfield hydrophone may have been off-axis (the dolphins could move their heads while in the hoop).

## Discussion

### Summary of pre-auditory wave (PAW) characteristics

In this study, electrophysiological and acoustical measurements were used to investigate the origin of the PAW in averaged EEGs of echolocating dolphins. The measurements revealed the PAW in both dolphins when epochs of instantaneous EEG data were temporally aligned with emitted clicks and averaged (Fig. 5). Visualization of individual epochs of EEG data showed that the PAW was likely present in each epoch (Fig. 6), and as each EEG epoch was associated with a single biosonar click, the PAW appears to be present with each emitted click. Furthermore, the relatively large amount of jitter in the dolphins’ click emissions (Fig. 3) means that the PAW must be temporally aligned (within less than ~ 1 to 2 ms) with the click to which the EEG epoch was aligned—i.e., if the PAW was the result of a previously emitted click, the jitter in click emissions would result in cancellation during the averaging process and the PAW would not be visible in the averaged EEG. Finally, the results of the present study suggest that the PAW amplitude may increase with click level, though these data should be treated with some caution given the small range of click levels recorded.

### Auditory origin for the pre-auditory wave (PAW)?

**Fig. 8:**
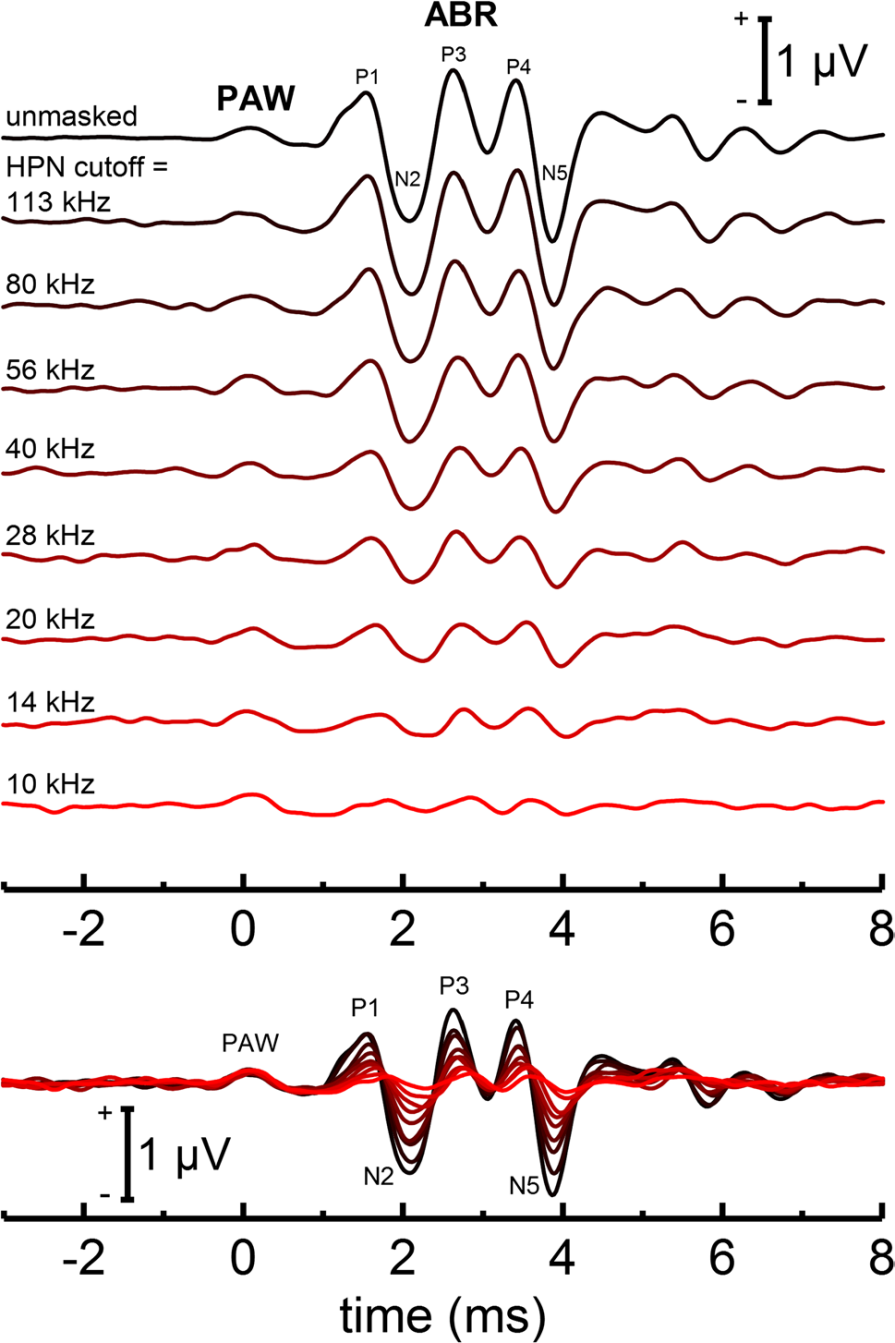
ABRs to self-heard biosonar clicks previously measured in SAY in the presence of masking noise with high-pass cutoff frequencies from 10 to 113 kHz. Data are from Finneran et al. (2017), with the time reference adjusted to the estimated time of click generation and the abscissa extended to − 3 ms. As the masking noise high-pass cutoff frequency was lowered, the ABR was progressively reduced in amplitude; however, the PAW amplitude did not change. This suggests a non-auditory origin for the PAW

In examining the origin of the PAW, an auditory source is first considered. PAW peak latencies were well-below those of ABR peak P1, which arises from primary neurons within the auditory nerve, ruling out neurogenic auditory evoked potentials (i.e., those arising from the auditory nerve and more distal sources along the ascending auditory pathway) as the origin of the PAW. Cochlear (i.e., receptor potential) origins of the PAW seem unlikely, given the particular electrode positions (far from the cochlea). Furthermore, previously published dolphin ABRs to self-heard clicks measured in the presence of high-pass masking noise (Fig. 8, Finneran et al. 2017) reveal that changes in noise high-pass cutoff frequency from 10 to 113 kHz had no effect on the PAW, but significant effects on the ABR to the self-heard click. Finally, the initial negative deflection for the PAW began up to 800 μs before the estimated time of click generation. Click generation time was estimated from the biosonar click arrival time measured at the center contact hydrophone, the distance from the hydrophone to the click generator (estimated to be 10 cm), and the sound speed (estimated as 1450 m/s). Errors associated with the distance and sound speed values were estimated to be no larger than ± 2 cm and ± 100 m/s, respectively, resulting in a maximum uncertainty in the estimated time of click generation of ± 20 μs. The error in the estimate of click emission time was therefore much less than the time difference between the onset of the PAW and click generation; i.e., errors in the estimate of click emission time cannot explain PAW deflections beginning before click generation. Thus, the PAW represents an electrophysiological signal that is time-locked to each emitted click but begins before click generation, is insensitive to acoustic masking noise, and has peak latencies much shorter than the latency of P1, the auditory nerve response. These characteristics rule out the acoustic biosonar click as the origin of the PAW; e.g., the PAW cannot be an auditory evoked response to the self-heard click.

In some bats, contraction of the middle ear muscles begins in synchrony with echolocation pulse emission, causing a transient loss of hearing sensitivity that recovers over time after pulse emission (Wever and Vernon 1961; Henson 1965; Suga and Jen 1975). Electromyograms of the activity of the stapedius and tensor tympani muscles in the little brown bat (*Myotis lucifugus*) showed activity beginning 4–6 ms before vocalization (Suga and Jen 1975). Rates of middle ear muscle contractions were measured up to 100/s and the tetanus-fusion frequency in the stapedius muscle was estimated to be 260–320/s (Suga and Jen 1975). This indicates the potential for electrophysiological signals to occur in synchrony with click emissions at high rates. However, the extent to odontocetes possess a middle ear reflex is unknown, and studies of receiver-based automatic gain control in echolocating odontocetes implicate a forward-masking based mechanism rather than a middle ear reflex (e.g., Supin et al. 2008). Spatial properties of the PAW also seem to rule out middle ear muscle activity: a previous study reported a decline in PAW amplitude as the non-inverting electrode was moved caudally away from the blowhole (Finneran et al. 2016). In the present study, PAW amplitudes progressively declined as the non-inverting electrode was moved laterally away from the blowhole at a fixed caudal distance. Changes to the PAW morphology with changes in electrode position were more substantial compared to those observed in the ABR. This suggests that the source of the PAW is relatively close to the skin surface (compared to ABR sources). The largest PAW amplitudes occurred when the non-inverting electrode was offset to the right of the midline; this side also coincided with the apparent side of biosonar click generation determined from click arrival times measured with contact hydrophones. Together, the temporal and spatial properties of the PAW suggest a source physically located near the blowhole (not the ear), offset towards the side from which clicks were produced.

### Myogenic origin for the pre-auditory wave (PAW)?

The temporal and spatial properties of the PAW suggest that it could be a neural or myogenic potential (or the superposition of both) related to muscle activity involved in biosonar click production, e.g., action potentials (APs) from motor neurons initiating click production or myogenic potentials arising from muscle contraction during click production (e.g., motor unit action potential, MUAP, or compound muscle action potential, CMAP). Since nasal cavity air pressure is required for pulse production (Ridgway et al. 1980; Amundin and Andersen 1983; Cranford et al. 2011), a muscular origin of the PAW may indicate that muscle action (producing an electrophysiological potential) is necessary to release air pressure in the production of each echolocation click.

**Fig. 9:**
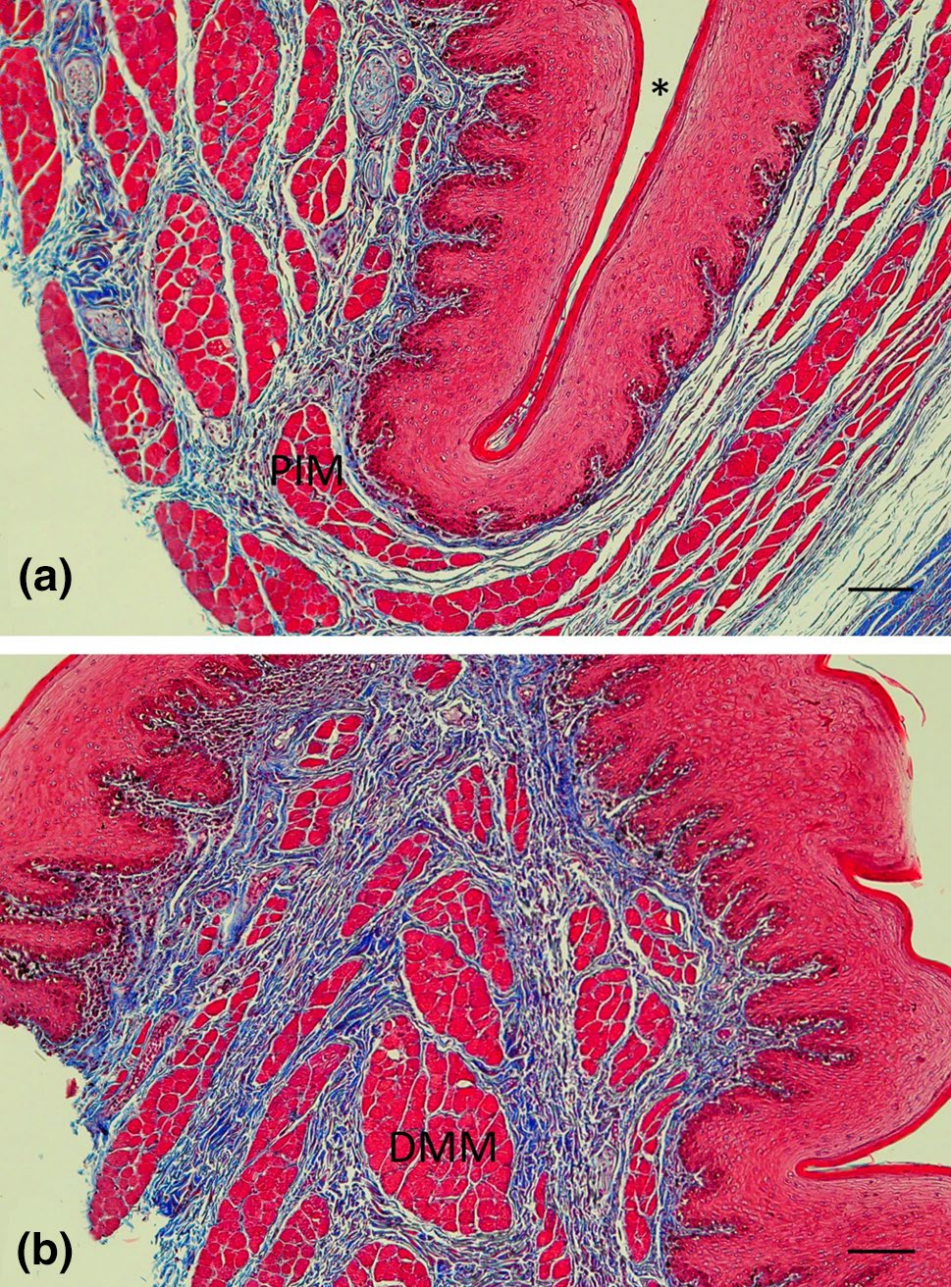
**a** Histological section of the region surrounding the posterior nasofrontal sac (*) showing the posterior internus muscle (PIM) wrapping around the sac (in cross section). **b** Histological section showing the diagonal membrane muscle (DMM) in cross section close to the skin surface. Sections are 5 μm-thick, stained with Masson’s Trichrome (muscle and epithelium in red, and connective and neural tissue in blue). Scale bar (bottom right in each panel) 100 μm

Several muscles exist in the vicinity of the nasal sacs/phonic lips and are therefore, probable sources of myogenic potentials during click production. A small complex of muscle fibers, the intrinsic muscles, surrounds both the anterior and posterior nasofrontal sacs (the only diverticula in delphinids with small intrinsic muscles) (Figs. 1a, 9a). Another small muscle, the diagonal membrane muscle (DMM), originates from the dorsolateral aspect of the vertex of the skull and has fibers that are oriented anteroventrally to insert on the margins of the paired diagonal membranes within the bony nasal cavity (Fig. 1b, 9b, Heyning and Mead 1990). The diagonal membrane muscle is the closest to the skin surface; however, the intrinsic muscles surrounding the posterior nasofrontal sac are also nearby (see Fig. 9a). Based on electromyogram measurements, Ridgway et al. (1980) reported that the DMM, as well as the anterior internus, the posterior internus, and the nasal plug muscle, was consistently involved in sound production. Thometz et al. (2017) reported that the nasal musculature surrounding *T. truncatus* left phonic lips (site of whistle generation) contained a greater percentage (58%) fast-twitch muscle fibers than the right nasal musculature (45%) (site of click generation) in three out of the four specimens. However, the left nasal musculature was found to contain a high concentration of myoglobin, which functions to deliver oxygen during intense muscle activity. These findings suggest that fast contraction of nasal muscles is possible, and might be a plausible source of the PAW. The fiber type analysis in Thometz et al. (2017) did not examine individual nasal muscles, and it is possible that the anterior internus, posterior internus, intermedius, anterior externus, posterior externus, and DMM, or any combination of these muscles might contain higher percentages of fast-twitch fibers, which may allow them to act before each click.

Electrophysiological potentials recorded with surface electrodes can vary in polarity depending on the orientation of the underlying sources relative to the recording electrodes (Dumitru and Jewett 1993), and both positive and negative waves may be recorded with surface electrodes from neural or myogenic sources. The multi-phasic waveform of the PAW could therefore, have resulted from either neural or myogenic sources (or both). However, the duration of the PAW (~ 0.6–0.7 ms for each wave, total duration ~ 2 ms) is on the order of those for peaks of the ABR, which arise from action potentials in nerve fibers, and is shorter than typical durations of myogenic potentials resulting from muscle contraction associated with multiple motor units (Picton 2011). MUAPs and CMAPs have longer durations than single fiber APs as a result of variation in arrival times for motor neuron AP at different endplates, jitter in neuromuscular synapses, and variation in muscle fiber propagation velocity (caused by different diameters) (Gootzen 1990). The short duration of the PAW—indicating a source with high synchrony—suggests either a neural source or a myogenic source with few motor units, short/consistent fiber length, and/or consistent propagation velocity.

Since the PAW was present in each epoch of EEG data delineated by a single click emission (Fig. 6), a muscular origin for the PAW implies muscle contraction associated with the generation of each individual biosonar click; i.e., that each individual click is under active neuromuscular control. Such muscular activity would require high contraction rates. For bats, experimental measurements have shown that each vocalization is under active neuromuscular control (Suthers and Fattu 1973) and calling rates are limited by contraction rates of “superfast” muscles with maximum rates ~ 180 to 200 Hz (Elemans et al. 2011). Fast muscle-contractile rates have also been reported in other species: e.g., muscles encircling the swimbladder of the toadfish (*Opsanus tau*) contract at rates up to ~ 200 Hz (Rome et al. 1996) and tetanus-fusion frequencies for the cat internal rectus muscle were described to be 240–300 Hz after stimulation of the horizontal canal nerve (Suzuki and Cohen 1966). Although most clicks in the present study were produced at repetition rates from ~ 10 to 30 Hz—below reported maximum muscle-contractile rates for other species, burst-pulse responses (i.e., rapid click sequences) from TRO had rates of ~ 300 Hz. Other studies have reported click rates for dolphins up to 430 Hz (Madsen et al. 2013), rates within burst-pulses up to 500–900 Hz (Blomqvist and Amundin 2004; Branstetter et al. 2012), and rates within terminal buzzes of 250–500 Hz (Wisniewska et al. 2014). Furthermore, evidence suggests that dolphin whistles may actually be formed via rapid production of clicks, meaning that click production rates may be as high as ~ 4–20 kHz (Madsen et al. 2012). At present, it is not known if muscles in the dolphin nasal system associated with click production can achieve the high contraction rates required to precede each individual click or whether they possess superfast characteristics. It is also possible that myogenic control of click production differs between generating echolocation clicks at relatively low rates (e.g., below ~ 100 Hz) and producing terminal buzzes, burst-pulses, or whistles, and that the PAW is not associated with individual clicks during the latter.

### Mechanoreceptor origin for pre-auditory wave (PAW)?

Prahl et al. (2009) reported the presence of mechanoreceptors in the connective tissue between the phonic lips and adjacent dorsal bursae in the harbor porpoise (*Phocoena phocoena*). Analogous structures have not been reported near the phonic lips in dolphins, but similar mechanoreceptors have been described in the skin (Palmer and Weddell 1964). Given the general similarities in sound production structures across odontocetes, it is not unreasonable to suppose that mechanoreceptors may also be present within connective tissue near the phonic lips in dolphins. If present, mechanoreceptors near the phonic lips may respond to pressure/vibration events associated with click production and produce time-locked evoked responses; however, it not known if evoked responses from mechanoreceptors could be detected with surface electrodes or if they could actually precede click emissions.

## Conclusions

A time-locked electrophysiological potential (the pre-auditory wave, PAW) precedes the production of individual biosonar clicks in bottlenose dolphins. Although the specific source of the PAW is unknown, the temporal and spatial properties of the PAW rule out an auditory system origin. The PAW may arise from muscle contraction associated with click production; however, it is not known if muscles within the dolphin nasal system can contract at the high rates typical of dolphin click production. It may be possible that the PAW originates from mechanoreceptors located near the phonic lips, but the extent to which mechanoreceptors exist within the dolphin nasal system and whether such mechanoreceptors could produce time-locked evoked responses preceding click emissions and detectable with surface electrodes are unknown. Measurements of the PAW at various click rates (e.g., normal echolocation to burst-pulse/buzzing rates) and over a broader range of click levels should shed additional light on the origins of the PAW. More detailed study of the arrangement and sub-cellular composition of the various muscles related to click production is also necessary to fully understand if dolphins can actively control the production of individual biosonar pulses.
